# Modelling the impact of Omicron and emerging variants on SARS-CoV-2 transmission and public health burden

**DOI:** 10.1038/s43856-022-00154-z

**Published:** 2022-07-25

**Authors:** Epke A. Le Rutte, Andrew J. Shattock, Nakul Chitnis, Sherrie L. Kelly, Melissa A. Penny

**Affiliations:** 1 Swiss Tropical and Public Health Institute, Allschwil, Switzerland; 2 University of Basel, Basel, Switzerland

**Keywords:** Epidemiology, Viral infection

## Abstract

**Background:**

SARS-CoV-2 variants of concern, such as Omicron (B.1.1.529), continue to emerge. Assessing the impact of their potential viral properties on the probability of future transmission dominance and public health burden is fundamental in guiding ongoing COVID-19 control strategies.

**Methods:**

With an individual-based transmission model, OpenCOVID, we simulated three viral properties; infectivity, severity, and immune-evading ability, all relative to the Delta variant, to identify thresholds for Omicron’s or any emerging VOC’s potential future dominance, impact on public health, and risk to health systems. We further identify for which combinations of viral properties current interventions would be sufficient to control transmission.

**Results:**

We show that, with first-generation SARS-CoV-2 vaccines and limited physical distancing in place, a VOC’s potential future dominance is primarily driven by its infectivity, which does not always lead to an increased public health burden. However, we also show that highly immune-evading variants that become dominant, even in the case of reduced variant severity, would likely require alternative measures to avoid strain on health systems, such as strengthened physical distancing measures, novel treatments, and second-generation vaccines. Expanded vaccination, that includes a booster dose for adults and child vaccination strategies, is projected to have the biggest public health benefit for a highly infective, highly severe VOC with low immune-evading capacity.

**Conclusions:**

These findings provide quantitative guidance to decision-makers at a critical time while Omicron’s properties are being assessed and preparedness for emerging VOCs is eminent. We emphasise the importance of both genomic and population epidemiological surveillance.

## Plain Language Summary

SARS-CoV-2 has mutated over time, resulting in new variants of the virus becoming the most common variant present in a population. The mutations that might alter characteristics of the virus, such as increasing the ability to infect people (infectivity), produce more severe illness (severity), or reduce the response of the immune system, are described as variants of concern (VOCs). We used a mathematical model of SARS-CoV-2 transmission to show that the infectivity of a future variant is the primary determinant of whether it will become most common, but that this increased ability to infect people does not always lead to an increased public health burden. Increasing vaccination is projected to have the biggest public health benefit for highly infective, severe variants that the immune system responds to. However, variants that do not respond to any previously gained immunity, even if less severe, would require alternative measures for control. These findings provide quantitative guidance when preparing for emerging VOCs.

## Abstract (web summary)

Le Rutte, Shattock et al. develop an individual-based SARS-CoV-2 transmission model to identify thresholds for dominance, health impact, risk to health systems, and required interventions for emerging variants of concern. This model shows that even if an emerging variant is less severe, first generation vaccines might not be sufficient to avoid overloaded health care systems.

## Introduction

SARS-CoV-2 has been mutating continuously since its emergence in December 2019, leading to viral variants with varying infectivity, severity, and immune-evading properties. On 26 November 2021, the World Health Organization identified Omicron (B.1.1.529) as a new variant of concern (VOC) after approximately seven months of Delta variant (B.1.617.2) dominating global transmission^1^. Within one week, 24 countries had reported cases of the Omicron variant^2^, with infections also occurring in previously infected and double-vaccinated individuals^3^. By mid-January 2022, >50% of all sequenced cases in Europe were caused by Omicron^4^, in which we include BA.2 as it is a sublineage of B.1.1.529. Whilst scientific research assessing the infectivity, severity, and immune-evading properties of Omicron has been ongoing, understanding the potential scope of the associated public health burden of Omicron and emerging VOCs is of top priority^5^.

In many European settings, high vaccination rates—particularly among those most at risk of severe disease—have led to a reduction in disease burden. However, protection of the most vulnerable and maintaining low levels of SARS-CoV-2 transmission in the northern hemisphere has been under threat at the time of Omicron’s emergence for multiple reasons. First, indoor contacts have been increasing during the cooler season. Second, immunity of the most vulnerable population (people 65+ and those with comorbidities) had started to wane since they were primarily vaccinated in the first quarter of 2021^6^. Last, relaxation of most non-pharmaceutical interventions (NPIs) since the summer of 2021 and fatigue of COVID-19 restrictions had led to increased contacts^7^. The emergence of Omicron in December 2021 combined with these threats called into question whether the vaccination strategies identified at the time would be sufficient.

Mathematical models have been used to represent transmission dynamics of SARS-CoV-2 and have supported decision-makers throughout the pandemic on the implementation and relaxation of control strategies^8–12^. Here we further developed and applied OpenCOVID, an individual-based model of SARS-CoV-2 transmission and COVID-19 disease, which includes seasonality patterns, waning immunity profiles, vaccination and NPI strategies, and properties for multiple variants^11^. We applied the OpenCOVID model to represent a general European setting and simulated the emergence of a novel variant. We analysed disease dynamics for a wide range of infectivity, severity, and immune evasion properties—relative to the Delta variant—representing a large range of potential properties for Omicron or for any other emerging VOCs. This allowed us to determine the potential for Omicron to become the new dominant variant, and to estimate its potential future public health burden. We further identified combinations of properties for which first-generation vaccines, in the absence of strong NPIs, would be sufficient to contain transmission and public health burden (i.e., new SARS-CoV-2 infections, hospital occupancy, and COVID-19-related deaths) and identified those combinations for which additional measures would be required.

We show that, with first-generation SARS-CoV-2 vaccines and limited physical distancing in place, a VOC’s potential future dominance is primarily driven by its infectivity. Future dominance does, however, not always lead to an increased public health burden. We also show that a highly immune-evading variant that becomes dominant, even in the case of reduced variant severity, would likely require alternative measures to first-generation vaccines to avoid strain on health systems, such as strengthened physical distancing measures, face mask usage, novel treatments, and second-generation vaccines. Expanded vaccination, which includes a booster dose for adults and child vaccination strategies, is projected to have the biggest public health benefit for a highly infective, highly severe VOCs with low immune-evading capacity.

## Methods

### OpenCOVID individual-based model

OpenCOVID is a stochastic, discrete-time, individual-based transmission model of SARS-CoV-2 infection and COVID-19 disease^11^. The model simulates viral transmission between infectious and susceptible individuals that come in contact through an age-structured, small-world network. The probability of transmission in each exposure is influenced by the infectiousness of the infected individual, the immunity of the susceptible individual (acquired through previous infection and/or vaccination), and a background seasonality pattern (reflecting a larger proportion of contacts being in closer contact indoors with cooler temperatures). Infectiousness is a function of viral variant infectivity and time since infection (which follows a gamma distribution peaking approximately at the time of symptom onset). Once infected, a latency period is followed by a pre-symptomatic stage, after which the individual can experience asymptomatic, mild, or severe infection. Severe cases can lead to hospitalisation, ICU admission, and ultimately death. Recovery after infection leads to the development of immunity. This immunity is assumed to wane over time and can be further reduced if exposed to a novel variant with immune-evading properties. The model has the capacity to represent a number of containment measures, including non-pharmaceutical interventions such as physical distancing and facemask usage, testing strategies such as test/diagnose isolate, mass-testing, and contact tracing, and also pharmaceutical interventions such as vaccination and treatment.

### Reproducibility

Detailed model descriptions and model equations are described in Shattock et al. (2022)^11^. Open access source-codes for the OpenCOVID model of all analyses presented in this study are publicly available at https://github.com/SwissTPH/OpenCOVID/tree/manuscript_december_2021/src, all figure code is also available at https://zenodo.org/record/6532404^13^.

### Vaccination

In this analysis, we simulate the impact of mRNA vaccines Pfizer/BioNTech and Moderna, which together make up 78% of the total number of doses secured in Europe at the time of writing^14^. Fully susceptible, partially susceptible, and infected individuals not in hospital are considered eligible to receive a vaccine. Vaccines have a two-fold effect; first, they provide protection against new infections through the development of immunity (90% reduced susceptibility). Second, once infected, vaccines reduce the probability of developing severe symptoms, leading to a 95% reduction in severe disease, impacting hospitalisations, ICU admissions, and deaths. We assume vaccination does not impact the probability of onward transmission once the individual is infected. Details regarding targeted vaccination groups and assumed durations between doses and vaccine efficacies are described in the Supplementary Methods sections 2.2 ‘Vaccine rollout’ and  2.3 ‘Vaccine-induced immunity profile’. Associated waning immunity profiles after infection and vaccination are described in Supplementary Methods sections 2.3 ‘Vaccine-induced immunity profile’ and 2.4 ‘Infection-induced immunity profile’, and Supplementary Fig. 1.

### Variant properties

Delta (B.1.617.2) is assumed to be the dominant transmission variant when Omicron (B.1.1.529) emerged. A full factorial range of Omicron properties was considered: 1) infectivity (transmission multiplication factor per exposure, relative to Delta), ranging from 0 to 2, 2) disease severity (multiplication factor per infection, relative to Delta ranging from 0 to 2), and 3) immune evasion capacity from 0 to 100%. In the model, once infected with the new variant, severity influences the chance to manifest severe symptoms, rather than experiencing mild or no symptoms. One hundred per cent immune evasion means fully evading any previously naturally or vaccination-acquired immunity, making an individual fully susceptible to infection with a new variant. The immune evasion property is assumed to only relate to the individual’s previously acquired immunity (their susceptibility), thus only impacting the rate of new infections and not influencing severity once infected. Full model details on the variant properties are described in Supplementary Information section 2.5 ‘Effect of variant properties and vaccination on prognosis’, alongside probabilities of immunologically naïve infected individuals developing the severe disease in Supplementary Table 1.

### Model initialisation

All model simulations were designed to be pseudo-representative of a European setting with simulations starting at the beginning of December 2021. We assume 30% of the population have been previously infected with SARS-CoV-2 over a 630-day period (representing an epidemic outbreak in Europe in March 2020). We assume the effective reproduction number (${R}_{e}\left(\tau \right)$) on 1 December 2021, is equal to 1.2. This represents an average scenario of increasing case numbers across Europe at the start of the winter period when the Omicron variant first emerged. This level of ${R}_{e}\left(\tau \right)$ is lower than levels in some European countries with strongly increasing cases as of early December, however it is higher than for those that implemented strong NPIs before December 1st. The average number of daily contacts required to achieve an ${R}_{e}\left(\tau \right)$ of 1.2 inherently considers any non-pharmaceutical interventions in place at the beginning of the winter period in Europe prior to the emergence of Omicron. Seasonality is assumed to follow a cosine function, with a peak in seasonal infectivity occurring 6 weeks from model initialisation (representing mid-winter, see Supplementary Fig. 2).

### Analyses

For the full range of variant properties specified, we simulated two vaccination scenarios from the introduction of the new variant to six months into the future. For the first scenario, no future vaccinations are implemented. The second scenario is identical up to 1 December 2021, but simulates expanded vaccination with first-generation vaccines administered as third-doses in adults (six months after the second-doses) and scale-up of first- and second-doses in 5-17-year-olds. Supplementary Table 2 provides the details of the two vaccination scenarios by risk group. Each simulation provided the relative prevalence of Omicron over the next 6 months compared with Delta, as well as daily and cumulative numbers of new SARS-CoV-2 infections, conservative maximum hospital occupancy, and COVID-19-related deaths. To reflect the element of chance that naturally occurs in transmission dynamics, 10 random stochastic simulations were performed per scenario for which we present the mean. In this analysis, we do not explore the effect of varying NPI intensity over time. As such, our analysis reflects predicted disease dynamics and public health burden in the absence of strong NPIs, such as lockdowns.

### Reporting summary

Further information on research design is available in the Nature Research Reporting Summary linked to this article.

## Results

### Disease dynamics projections

We explored a wide range of potential infectivity and severity levels (between zero and two) for the new Omicron variant (or any other emerging VOC) relative to Delta, and the full range of immune-evading capacity from 0% (similar to Delta) to 100% (no protection from current vaccines and previously acquired natural immunity following infection). Disease dynamics were simulated over a six-month period beginning 1 December 2021 when Omicron emerged, and the cooler weather arrived. We assume an initial effective reproduction number of 1.2, the absence of strict NPIs (e.g., lockdowns), and vaccination coverage by risk group as described in Supplementary Table 2.

We compared two future vaccination scenarios. First, expanded vaccination, with the administration of a third-dose six months after the second dose for individuals older than 12 years of age, and administration of vaccine doses 1 and 2 for children aged 5-11 years. Second, we simulated a scenario with no future vaccine rollout (no third-dose vaccination in over 12-year-olds and no vaccination of 5-11-year-olds). The latter leads to the decay of population-level vaccine-induced immunity over time. Seven combinations of variant properties and the associated variant’s probability to become dominant, the predicted public health burden, and the expanded vaccination effect are summarised in Supplementary Table 3 and visualized in Supplementary Figs. 3 and 4.

### Drivers of Omicron’s potential dominance

**Fig. 1:**
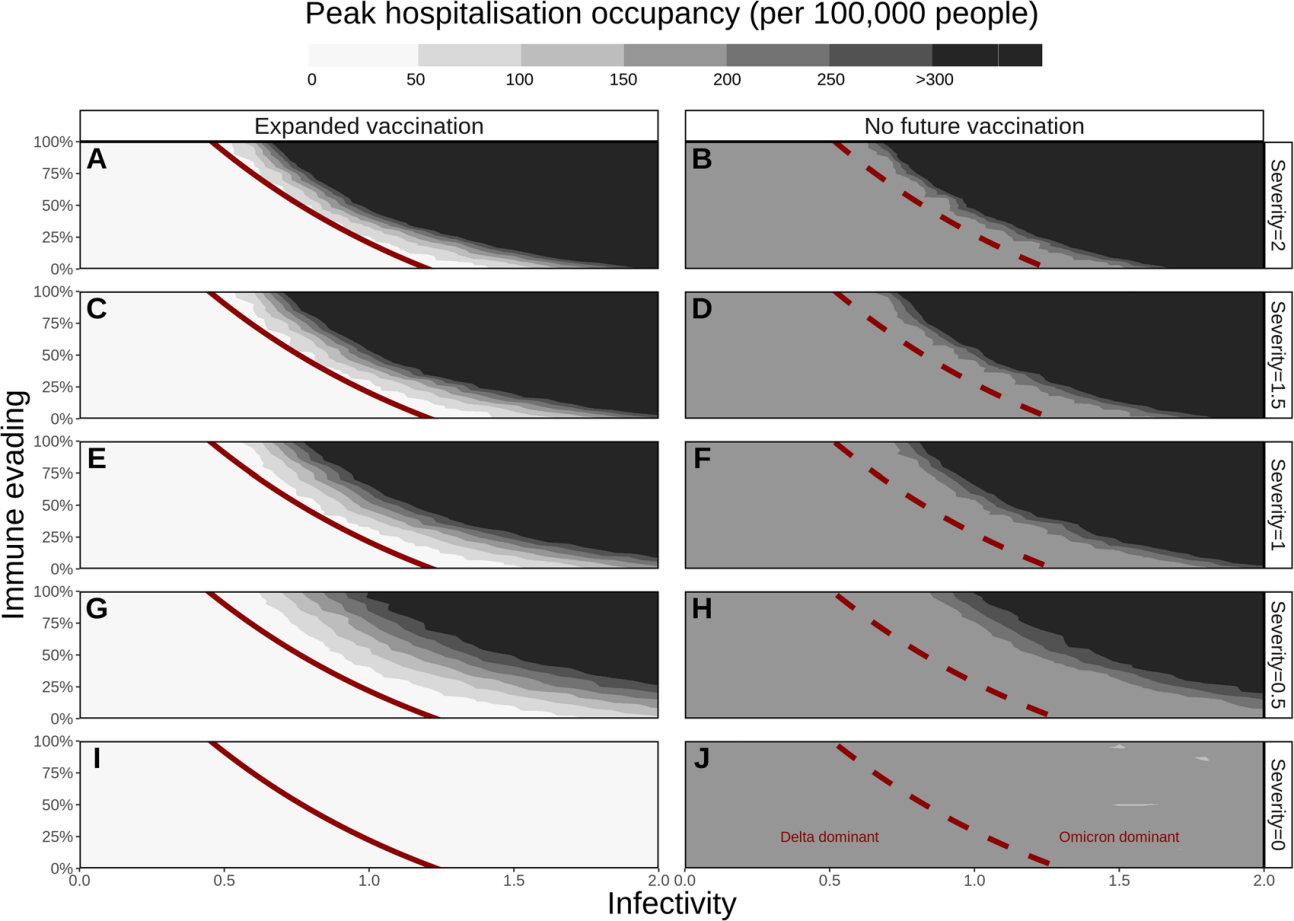
Peak daily hospital occupancy for a range of variant properties (number of beds per 100,000 population over the six-month simulation period). The threshold (50% prevalence, see Supplementary Fig. 5) for Omicron’s dominance is presented by red lines (solid for expanded vaccination in panels **A**, **C**, **E**, **G**, and **I**, dashed for no future vaccination in panels **B**, **D**, **F**, **H**, and **J**). Expanded vaccination includes third doses for adults (6 months after the second dose) and vaccinating 5-11-year-olds. Rows represent Omicron’s potential severity (0 to 2) relative to Delta (1). Horizontal axes represent the range of Omicron’s potential infectivity (0 to 2) relative to Delta (1), left vertical axes represent Omicron’s potential immune-evading capacity (0 to 100%). The peak hospital occupancy per 100,000 people has been conservatively capped at 300 per 100,000, a value that ranges for European countries between 220 (Sweden) and 800 (Germany)^16^.

**Fig. 2:**
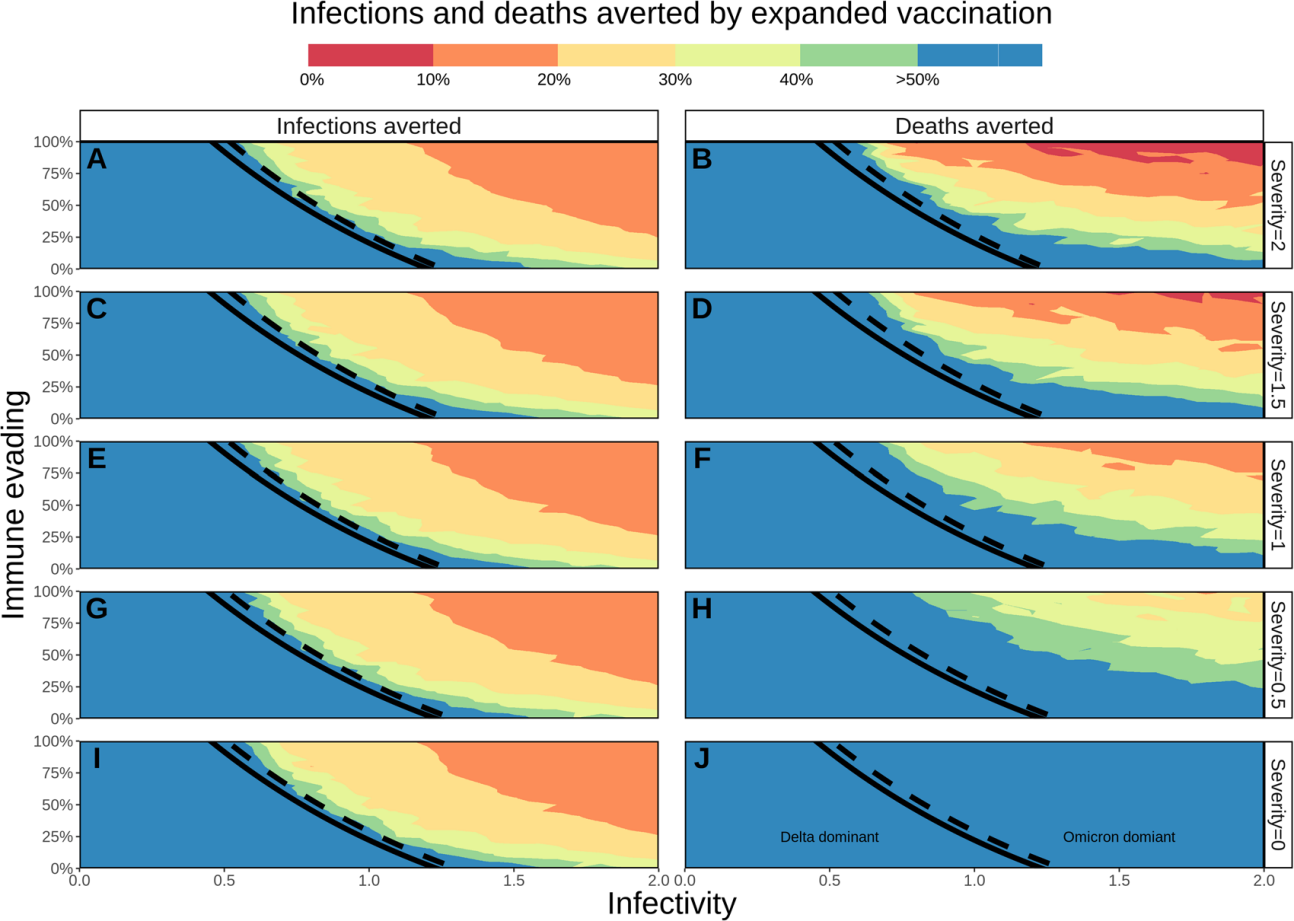
Percentage of COVID-19 infections and deaths averted by third-dose vaccines for adults and vaccinating 5–11-year-olds with doses one and two. The threshold (50% prevalence) for Omicron’s future dominance within 6 months is presented by black lines (solid line for expanded vaccination, dashed for no future vaccination). **A**, **C**, **E**, **G**, and **I** represent the percentage of infections averted, **B**, **D**, **F**, **H**, and **J** the percentage of deaths averted due to expanded vaccination, with colour representing % averted. Rows represent Omicron’s potential severity (0 to 2) relative to Delta (1), horizontal axes represent Omicron’s potential infectivity (0 to 2) relative to Delta (1) and left vertical axes represent Omicron’s potential immune-evading capacity (0 to 100%). The maximum percentage of burden averted is capped at 50%, with the blue area extending to values >50%.

Infectivity and immune evasion were identified as the drivers behind Omicron’s potential dominance, with negligible effect from increasing severity. For an infectivity factor of 1, a 25% immune evasion property was predicted to be sufficient for dominance within six months, while for 100% immune evasion an infectivity factor of 0.5 (half the infectivity of Delta) was predicted to be sufficient. All combinations of properties and their impact on future variant dominance are presented in Supplementary Fig. 5 and by the red solid and dashed lines in Figs. 1 and 2. When highly immune-evading, Omicron’s chance to dominate would even be slightly increased with future expanded vaccination, as first-generation vaccines provide a high level of protection against Delta variant infection^15^, consequently increasing the relative susceptibility of the population to a highly immune-evading Omicron infection.

### Disease burden

If Omicron or any other emerging VOC would have been less infective than Delta (below 1), with no immune-evading capacity, it would likely not become dominant and the public health impact due to its emergence would be negligible (Supplementary Fig. 5). Assuming Omicron or any other emerging VOC would have had a partial immune-evading capacity of 20% and be 20% more infectious than Delta, we predicted it would have taken approximately four months for it to become dominant. Although becoming the dominant variant, we predicted that these properties would lead to a relatively low increase in public health burden (as is illustrated by the area to the right of the red curves in Fig. 1 and Supplementary Figs. 6 and 7). However, in scenarios where Omicron, or any other emerging variant, would have had higher levels of immune evasion capacity (50%), increased infectivity of 50%, and be equally or more severe than Delta, dominance would occur within five weeks, resulting in a higher projected public health burden.

### Projected impact on peak hospital occupancy

Expanded vaccination was predicted to be sufficient to prevent high hospital occupancy (dark grey/black area of Fig. 1, capped at 300 hospital beds per 100,000 people) for settings in which Delta would remain dominant^16^. For a highly infectious (infectivity factor >1.5) but a non-immune-evading variant, we predicted that expanded vaccination would be sufficient to prevent high hospital occupancy, however, this probability diminished with increasing severity. Moreover, we explored a range of infectivity and immune-evading properties for which Omicron would become dominant, but was not expected to lead to high peak hospital occupancy under the assumption of expanded vaccination (white area on the right side of the red curve in Fig. 1 panels A, C, E, G, and I). Similarly, this range diminished with increasing severity. For a variant with infectivity and severity similar to Delta, we predicted that expanded vaccination would be insufficient to prevent high peak hospital occupancy for an immune-evading property above 75%. For properties that are predicted to lead to high hospital occupancy, settings would benefit from additional measures such as physical distancing, face mask usage, or new treatments and next-generation vaccines as they become available.

### Impact on COVID-19 infections and deaths

Omicron’s potential range of severity levels was found to have negligible influence on new infections (Supplementary Fig. 6); however, it would heavily influence future mortality (Supplementary Fig. 7) and maximum hospital occupancy would likely be reached. These simulations are in the absence of additional measures that would likely be implemented before such high rates of hospitalisation and mortality are reached. Figure 2 presents the percentage of infections and deaths averted through expanded vaccinations for all specified combinations of properties. The highest public health burden in terms of infections and deaths (top right corner of Supplementary Figs. 6 and 7), would be least likely to be reduced by expanded vaccination (top right of panels in Fig. 2), because of its highly immune-evading capacity. However, with Omicron becoming the dominant variant, expanded vaccination was predicted to have an impact on deaths for combinations of properties where severity was lower than for Delta (severity of 0.5 and 0.0 (Fig. 2)). The benefit of expanding vaccination will also be seen for scenarios where Delta remains dominant (area to the left of the black lines in Fig. 2).

**Fig. 3:**
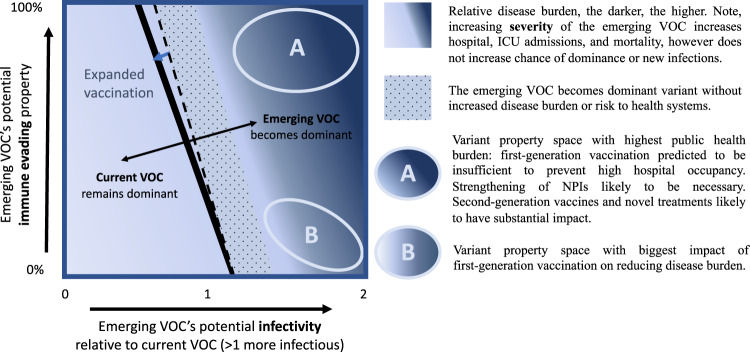
Schematic summary illustrating the interplay between potential infectivity, immune evasion of Omicron or another variant of concern on its chances of becoming the dominant variant and associated public health burden. The background colour represents the relative disease burden, the darker, the higher the burden. The solid black line represents the cut-off for which the emerging VOC does (right of the line) or does not (left of the line) become the dominant variant. The dotted area represents the parameter space for which the emerging VOC becomes dominant, but without increased disease burden or risk to health systems. Variant property space (**A**) represents the combination of the emerging VOC’s infectivity and immune-evading capacity for which the highest public health burden can be expected. Variant property space (**B**) represents the combination of the emerging VOC’s infectivity and immune-evading capacity where first-generation vaccines will have the biggest impact in reducing disease burden.

To aid in the interpretation of Omicron’s or any future VOCs properties on its chance of becoming dominant and the associated overall impact on public health burden in a vaccinated population, we summarised our findings in a schematic (Fig. 3). The darkness of colour in the heat map indicates the level of disease burden (infections and deaths) and risk to health systems (hospital occupancy) at the corresponding level of relative infectivity and immune evasion. The dashed line indicates when the emerging variant would become the dominant variant without future vaccinations, the solid line when it would become dominant with expanded vaccination. The dotted area indicates when the emerging variant would become dominant without increased disease burden or risk to health systems. Area A indicates variant property space where expanded vaccination alone will not prevent increased transmission and would lead to increased infections, hospital occupancy, and deaths. Additional measures would be needed in the case of a very high immune-evading variant including  once second-generation vaccines and/or novel treatment become available, or strengthened NPIs in their absence. Area B indicates space where expanded vaccination with first-generation vaccines will have the highest impact on reducing disease burden and health system risk. In case of low severity in area B, expanded vaccinations may be sufficient for control.

## Discussion

Using our OpenCOVID individual-based transmission model, we simulated the interplay of Omicron’s or any emerging VOC’s potential combinations of infectivity, severity, and immune-evading properties. We first identified the threshold for the emerging variant becoming the dominant variant (over Delta), for which infectivity was found to be the main driver followed by immune evasion. Increased disease severity of the emerging variant was found to have a limited effect on dominance, but when dominant, to allow for an increase in hospital occupancy and number of deaths. If the emerging variant has increased infectivity and severity to Delta, then expanding first-generation vaccination with a third-dose and vaccinating 5-11-year-olds could avert many cases and deaths. However, should the emerging variant be highly immune-evading, first-generation vaccines would not suffice, and additional measures would be required to control transmission until vaccines are updated.

Besides additional preventive measures such as strengthened NPIs and second-generation vaccines, new treatments that were approved in December 2021 can now additionally be implemented to reduce the public health burden^17–23^. Maximum hospital capacity will have to be considered as it differs between countries, ranging from 220 to 800 beds per 100,000-population capita in Europe^16^. In a worst-case scenario of highly immune-evading and increased severity of emerging variants, for many countries, our predictions reached or surpassed our assumed overall maximum hospital capacity. However, very high occupancy rates are unlikely to be reached as countries would implement additional measures sooner.

Although our analysis focuses on hospital occupancy and mortality as higher priority public health risks, even rising SARS-CoV-2 infections due to lower variant severity may lead to a substantial risk of increased long-COVID^24^ and should not be neglected in response planning. Furthermore, as we considered expanding vaccination with vaccination booster doses, the global inequity of vaccine access, and the selective pressure and emergence of new variants in settings with low vaccination rates is a global health emergency that needs to be addressed^25^.

Our transmission model is based on assumptions that influence its outcomes. In our model predictions, we identified that the emerging variant’s potential severity had little effect on the number of new cases, also because no impact on viral load was assumed. Higher viral load in the model is associated with increased transmissibility. The two vaccination scenarios we present in this study, expanded vaccination (with a third-dose for those 12 years of age and older, and two-dose vaccines for children aged 5–11 years) and no future vaccination, are examples of two extreme scenarios. The reality will lie in between and will be region dependent. Waning immunity is assumed to reduce linearly to zero in approximately one year both after vaccination and infection. However, should immunity wane much slower, then the population would be protected for a longer period against either Delta (or a new non-immune-evading variant), and our outcomes would prove too pessimistic. Our outcomes would be too optimistic should immunity wane faster than assumed. A higher or lower value of 1.2 for the effective reproduction number at the start of the winter period, prior to the introduction of Omicron, could have influenced peak hospital occupancy estimates, however, the relative impact of vaccination is not sensitive to this parameter.

With Omicron being more infectious, less severe, and slightly immune-evading relative to Delta, our model predicted that Omicron would indeed quickly become the dominant variant (within one to four months, depending on its exact combination of viral property values), leading to a peak in cases and hospitalisations (up to 50 hospital beds per 100,000 people with expanded vaccination). For these scenarios, we predicted that the expanded vaccination would prevent greater than 40% of hospitalisations. In European countries with an expanded vaccination rollout, the hospital occupancy peak during the Omicron outbreak ranged between 20 and 50 beds per 100,000 population, echoing our simulations^26^.

SARS-Cov-2 mutations have been identified throughout the pandemic and new variants are likely to continue to emerge. Compared with the Alpha variant, Delta has severity ratios reported as 1.3- to 2.3-times higher for hospital admissions and deaths^27^. Both vaccine-induced and naturally acquired immunity will not suffice to prevent infection or disease if a new variant is highly immune-evading. Based on these predictions, we stress that vaccines may need adjustment to respond to current or future viral mutations. We further advocate for continued genomic surveillance and see it crucial to quickly test and continue improving assays that elucidate immune evasion properties of a new VOC, to better understand the risk of increased infectivity, severity, and immune evasion.

In conclusion, as the properties of Omicron and any future SARS-CoV-2 VOCs become known, our analyses elucidate the interpretation of the variant’s potential dominance and subsequent public health burden. Combined with VOC genomic^28^ and population epidemiological surveillance^29^, alongside antibody neutralisation testing^30^, our findings provide crucial quantitative guidance to decision-makers at a critical time.

## Supplementary information

Supplementary Information

Peer Review File

Reporting Summary

## Data Availability

Data sharing is not applicable to this article as no data sets were generated or analysed during the study. Data informing model parameters are described in Shattock et al. (2022)^11^. Model output underlying Figs. 1 and 2 are provided as Supplementary Data via https://zenodo.org/record/6655746#.YqxAYuzMKda.

## Code availability

All model and figure code are open access and publicly available at https://github.com/SwissTPH/OpenCOVID/tree/manuscript_december_2021/src and https://zenodo.org/record/6532404^13^. The model and figure code for this study uses version 2.0 of OpenCOVID.
