# Supplementary material for: Modelling the impact of Omicron and emerging variants on SARS-CoV-2 transmission and public health burden
Source: Commun Med (Lond). 2022 Jul 25;2:93. doi: 10.1038/s43856-022-00154-z (PMC9311342; doi:10.1038/s43856-022-00154-z)
Supplement: Supplementary file 1 — Supplementary Information [file 43856_2022_154_MOESM1_ESM.pdf]

Supplementary Information

Modelling the impact of Omicron and emerging variants on SARS-CoV-2 transmission and public health burden

Epke A Le Rutte* [1,2], Andrew J Shattock* [1,2], Nakul Chitnis [1,2], Sherrie L Kelly [1,2], Melissa A Penny [1,2]

[1] Swiss Tropical and Public Health Institute, Basel, Switzerland

[2] University of Basel, Basel, Switzerland

* These authors contributed equally to this work

Correspondence to Prof Melissa A Penny

melissa.penny@unibas.ch

1. Supplementary Figures 1-7[1] and Tables 1-3

Supplementary Table 1. Probabilities that immunologically naïve (i.e., unvaccinated and previously uninfected) individuals with no comorbidities infected with Delta (severity factor 1, 0% immune evading) develop severe disease (source[4–7]).

Age group	Severe disease (Delta, unvaccinated)
0-10	<1.0%
10-20	1.0%
20-30	1.2%
30-40	2.4%
40-50	3.9%
50-60	6.6%
60-70	16.3%
70-80	26.0%
80-90+	33.0%

Supplementary Table 2. Overview of two vaccination scenarios. No future vaccination, and expanded vaccination through third-dose in adults (six months after second-dose) and scale up in 5-17-year-olds with first-generation vaccines. Additional details are provided in section 2.2 'Vaccine rollout'.

Group	As of 1 December 2021	No future vaccination	Expanded vaccination
65+ or comborbidities	90% coverage	90% coverage	90% coverage of third dose* 180 days after second dose
Adults 18-64	70% coverage	70% coverage	70% coverage of third dose* 180 days after second dose
Adolescents 12-17	50% coverage	50% coverage	Scale up to 70% coverage within 2 months
Children 5-11	0% coverage	0% coverage	Scale up to 50% coverage within 4 months
Total number of doses in future		zero	16,000 per 100,000 people over six-months

*5% dropout included between those receiving second and third-dose (95% acceptance rate for third dose for these with two doses).

Supplementary Table 3. Summary of seven combinations of Omicron's or any future variant of concern's potential properties including infectivity, severity, and immune evasion. Probability of the new variant to become the dominant variant, associated public health burden, and the effect of expanded vaccination through third-dose in adults (six months after second-dose) and scale up in 5-17-year-olds with first-generation vaccines. Effects on infections, hospitalisations, and variant dominance are visualized in Supplementary Figures 3 and 4.

Simulation	Infectivity (relative to Delta)	Severity (relative to Delta)	Immune evasion	Probability of Omicron dominance	Public health impact	Expanded vaccination* effect
1	1	1	0%	Low	Negligible	High
2	2	1	0%	Very high	High	High
3	2	2	0%	Very high	High	Very high
4	1	1	100%	Very high	High	Moderate
5	1.2	2	20%	High	Low	Moderate
6	1.5	2	50%	Very high	High	Low
7	2	2	100%	Very high	Very high	Negligible

* Expanded vaccination through third-dose in adults and vaccinating 5-17-year-olds with first-generation vaccines.

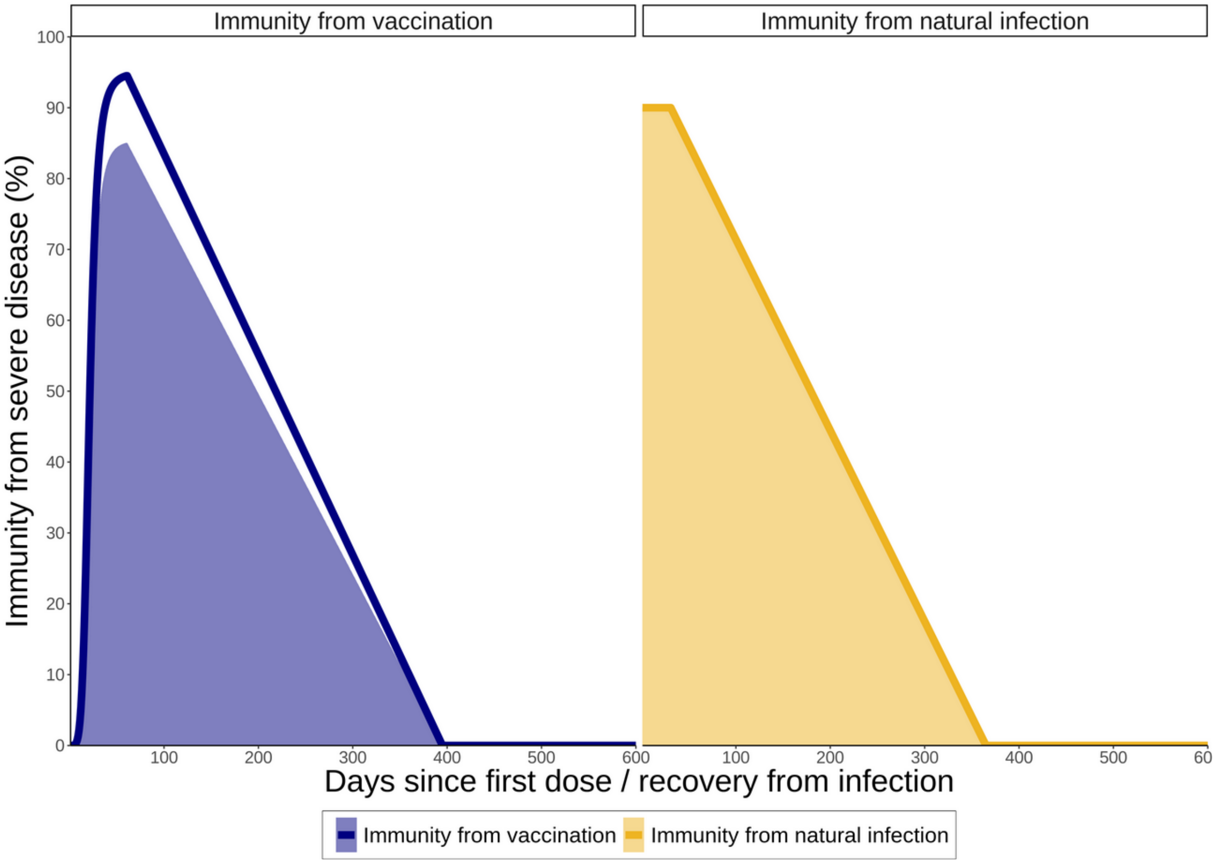

Supplementary Figure 1. Profiles for immunity acquired from a) vaccination, and b) natural infection. The shaded areas represent immunity from transmission when exposed to a variant without immune escaping properties. The solid line represents overall immunity to severe disease.

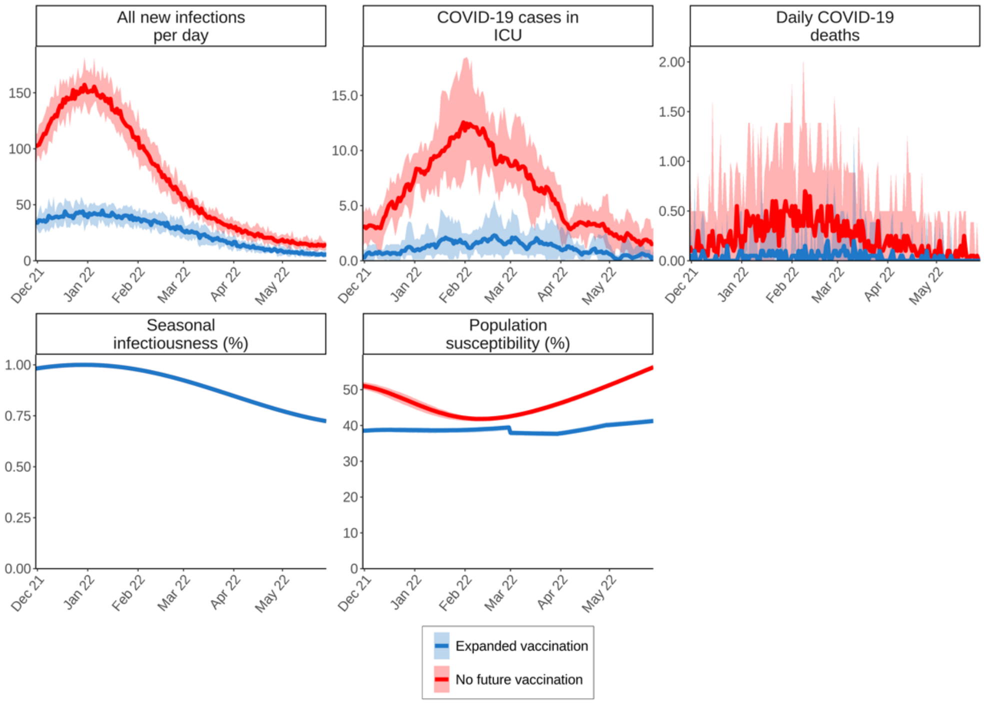

Supplementary Figure 2. Predicted temporal epidemiological trends for Delta in the absence of Omicron under no future vaccination and expanded vaccination, alongside seasonality profile. Public health burden is represented by COVID-19 infections, ICU occupancy and COVID-19 related mortality per 100,000 population per day. Seasonal infectious and susceptible population over time is presented in the bottom row. Blue illustrates dynamics with expanded vaccination, and red with no future vaccination. The shaded area around the predictions represents stochastic variation representing 10 separate simulations.

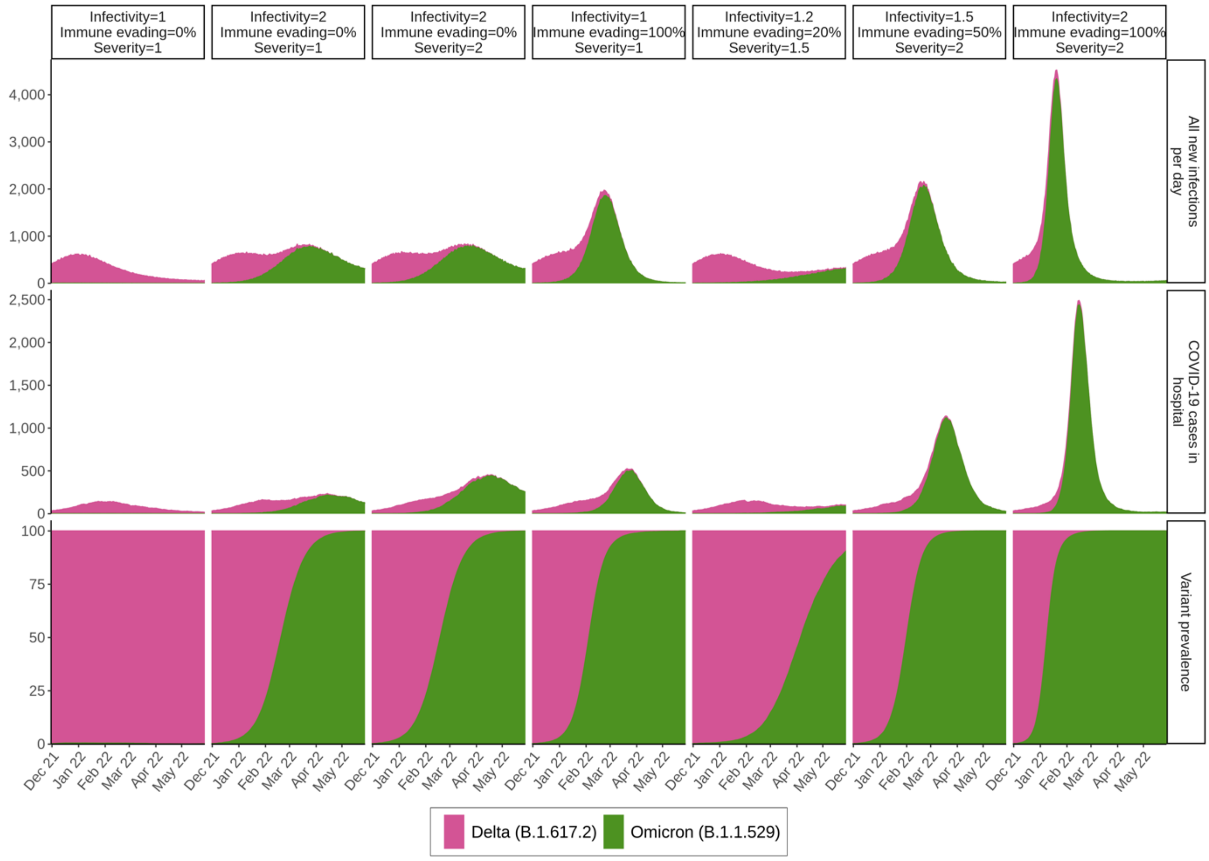

Supplementary Figure 3. Temporal epidemiological trends for seven combinations of the new variant's potential properties (see Supplementary Table 3) under no future vaccination. Public health burden is represented by COVID-19 infections and hospital occupancy per 100,000 population per day. Variant prevalence percentage over time is presented in the bottom row.

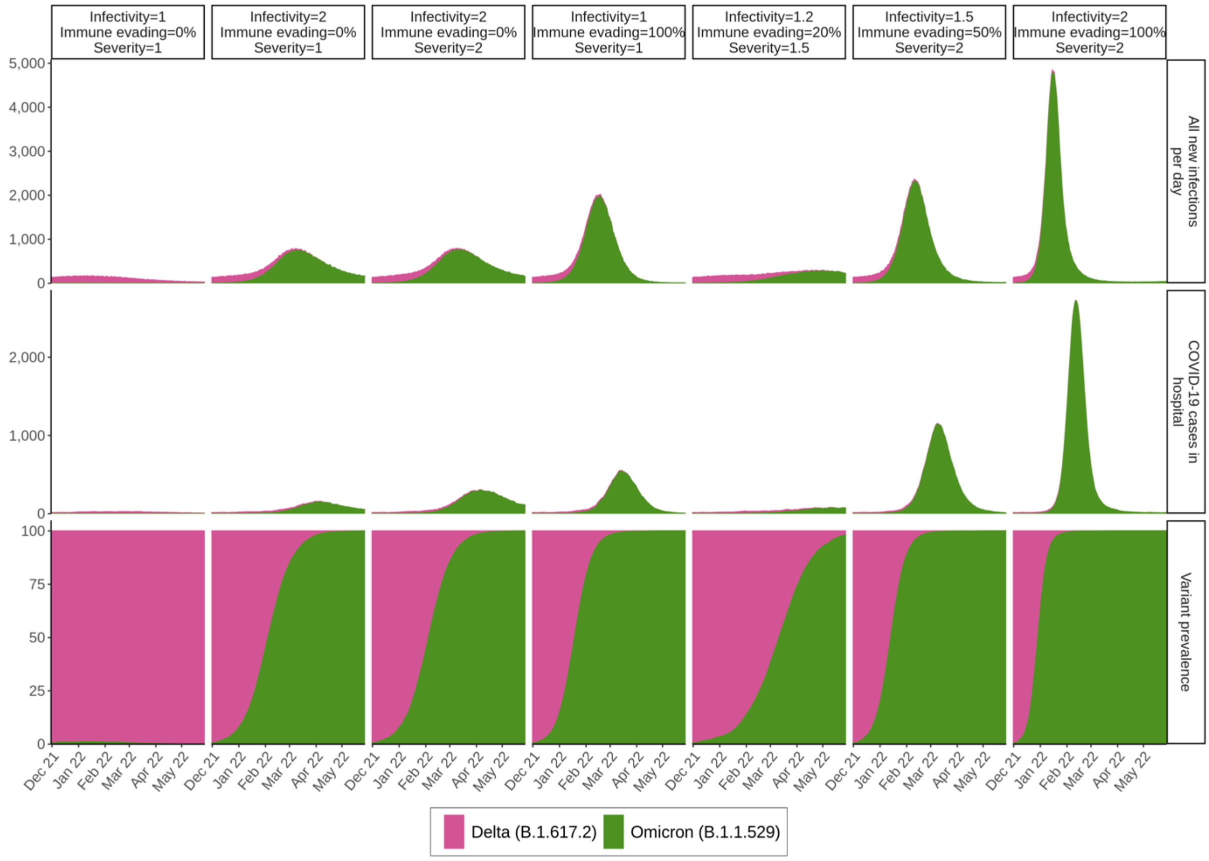

Supplementary Figure 4. Temporal epidemiological trends for seven different combinations of Omicron's potential properties (see Supplementary Table 3) under expanded vaccination. This figure represents a setting with future expanded vaccination through third-dose in adults (six months after second-dose) and scale up in 5-11-year-olds with first-generation vaccines. Public health burden is represented by COVID-19 infections and hospital occupancy per 100,000 population per day. Variant prevalence percentage over time is presented in the bottom row.

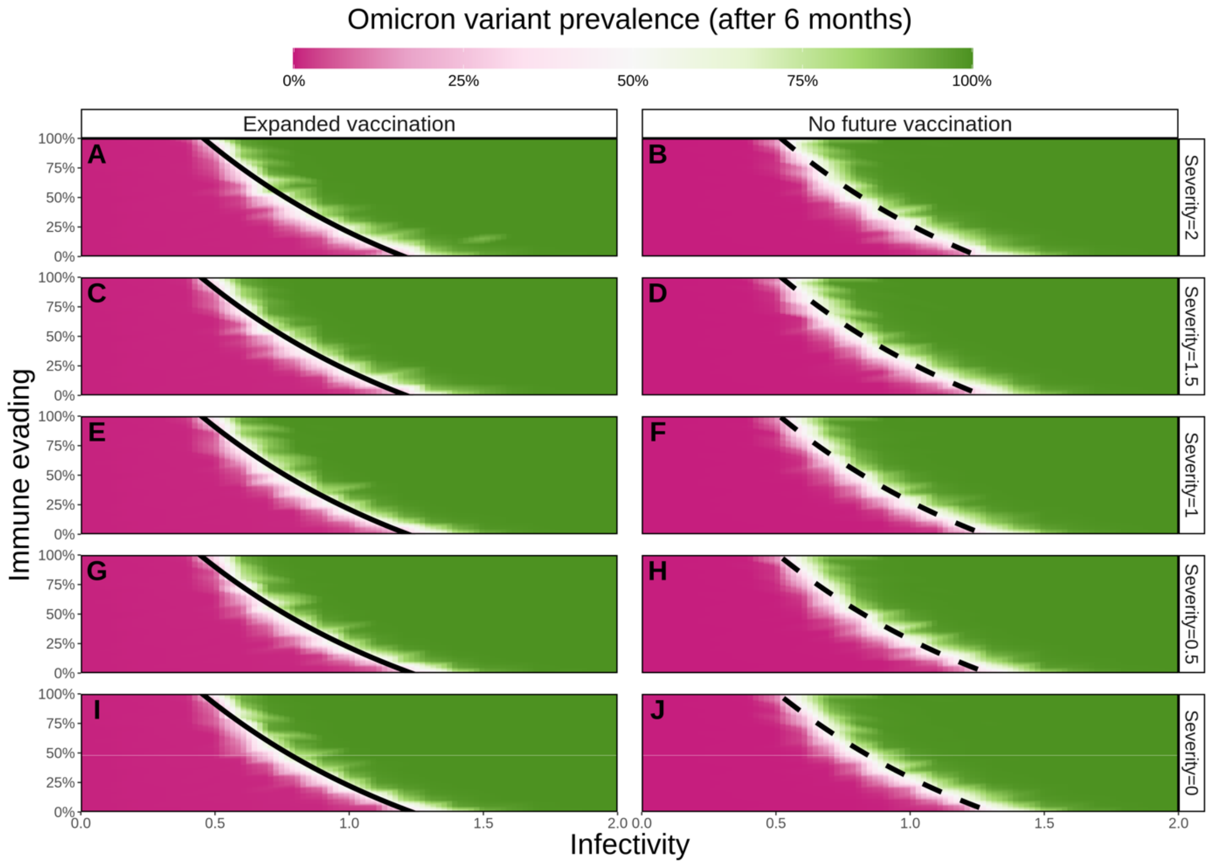

Supplementary Figure 5. The projected prevalence of the new variant under combinations of infectivity, immune evasion capacity, and severity after six months. Black line represents the threshold (50%) for the new variant to become dominant (areas to the right of the lines). Left column represents a setting with expanded vaccination through third-dose in adults (six months after second-dose) and scale up in 5-17-year-olds with first-generation vaccines. Right column represents a setting with no future vaccination. Horizontal axes represent the range of the new variant's potential infectivity (0 to 2) relative to Delta (1). Vertical axes represent the range of the new variant's potential immune evasion capacity (0 to 100%). Rows represent five levels of the new variant's potential severity (0 to 2) relative to Delta (1).

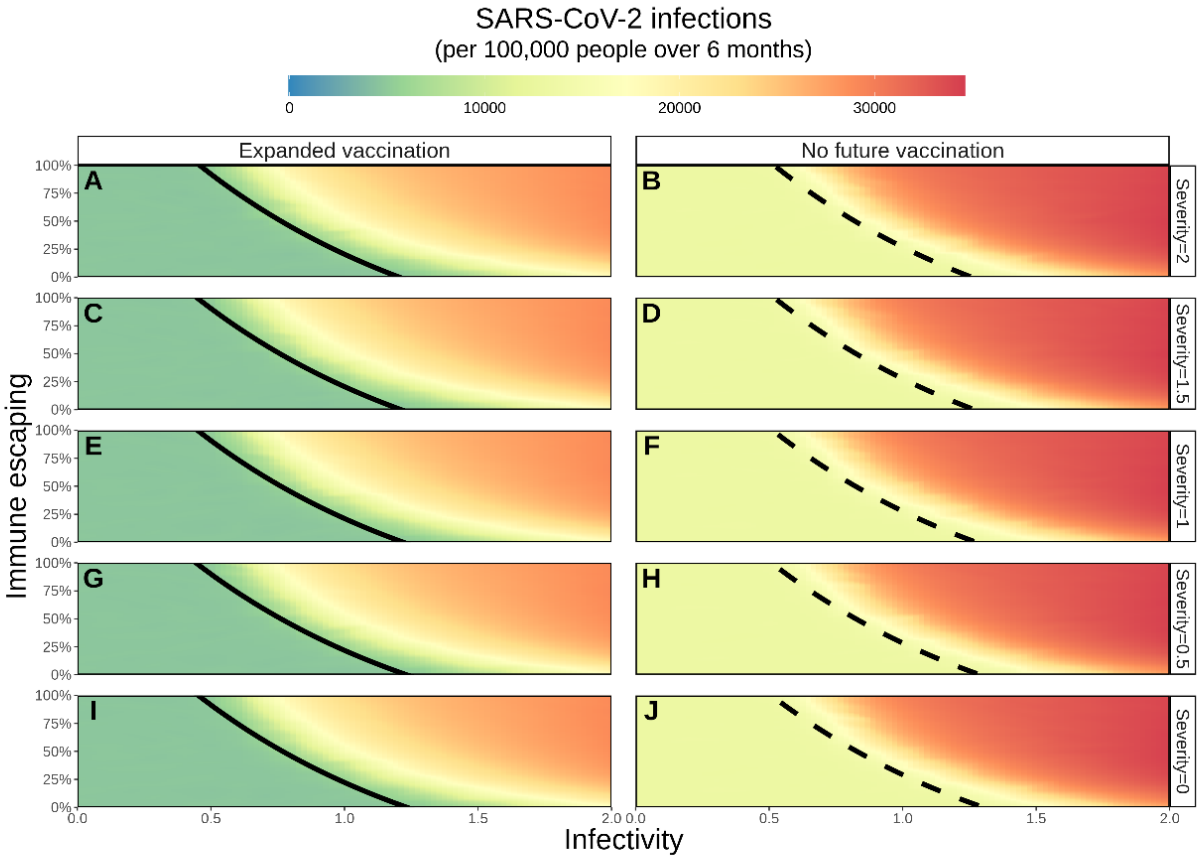

Supplementary Figure 6. Cumulative number of SARS-CoV-2 infections (per 100,000 population over the six-month simulation period) for a wide range of variant properties. Solid and dashed black lines represent the threshold (50%) for the new variant to become dominant (area to the right of the black line). Left panels represent a setting with expanded vaccination with first-generation vaccines through third-dose in adults (six months after second-dose) and scale up in 5-17-year-olds, right panels, a setting with no future vaccination. Horizontal axes represent the range of the new variant's potential infectivity (0 to 2) relative to Delta (1). Left vertical axes represent the range of the new variant's potential immune evasion capacity (0 to 100%). Rows represent five levels of the new variant's potential severity (0 to 2) relative to Delta (1).

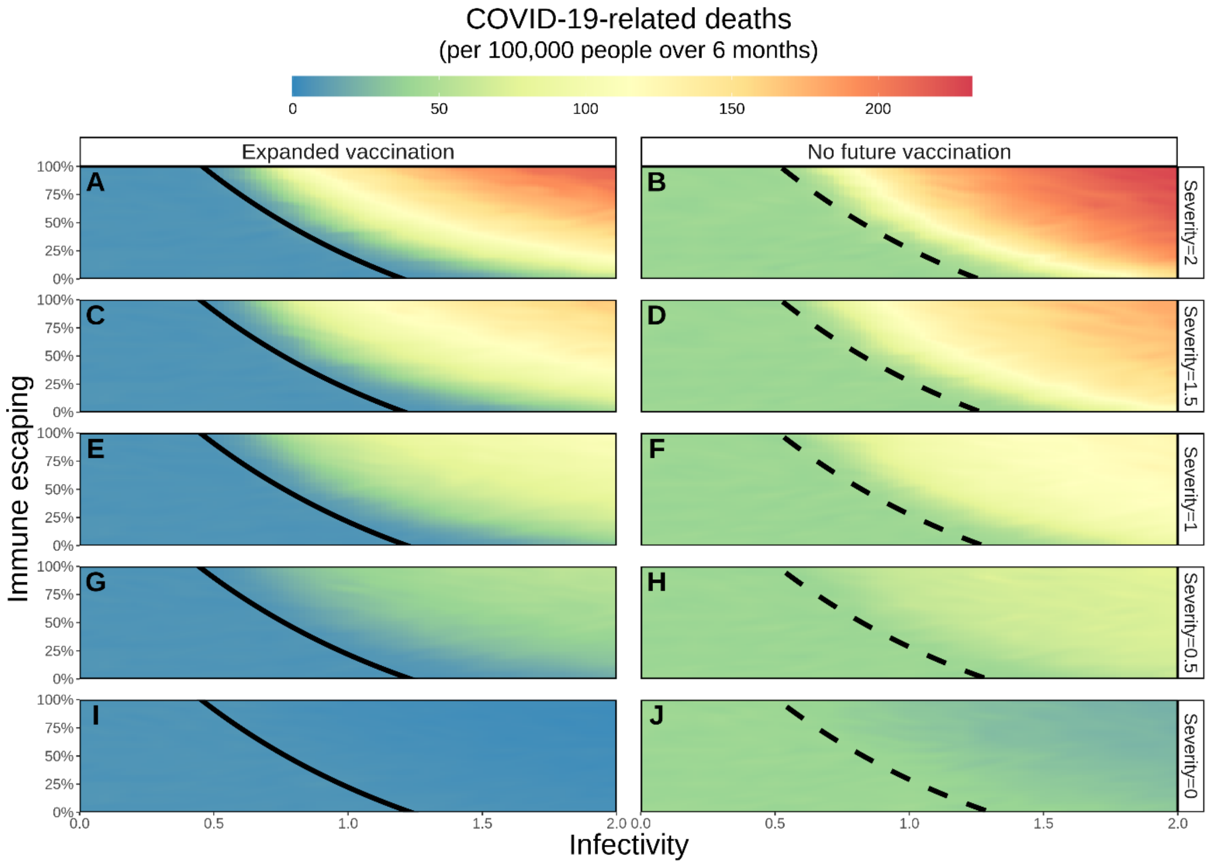

Supplementary Figure 7. Cumulative number of COVID-19-related deaths (per 100,000 population over the six-month simulation period) for a wide range of variant properties. Solid and dashed black lines represent the threshold (50%) for the new variant to become dominant (area to the right of the black line). Left panels represent a setting with expanded vaccination through third-dose in adults (six months after second-dose) and scale up in 5-17-year-olds using first-generation vaccines, right panels, a setting with no future vaccination. Horizontal axes represent the range of the new variant's potential infectivity (0 to 2) relative to Delta (1). Left vertical axes represent the range of the new variant's potential immune evasion capacity (0 to 100%). Rows represent five levels of the new variant's potential severity (0 to 2) relative to Delta (1).

2. Supplementary Methods

2.1 Model initialisation

All model simulations were designed to be pseudo-representative of a general Western European setting at the beginning of December 2021. We assume 30% of the population have been previously infected with SARS-CoV-2 over a 630-day period (representing epidemic outbreak in Europe in March 2020). The fraction that was both previously infected and

vaccinated prior to the emergence of the Omicron variant was age-dependent and ranged from 3% in 10-20-year-olds to 30% in 80-90-year-olds (see section 2.2 ‘Vaccine rollout’ for further details on vaccination rates prior to the Omicron’s emergence). We assume the effective reproduction number on 1 December 2021 is equal to 1.2. This represents increasing case numbers across Europe at the start of the winter period, prior to the emergence of Omicron. The average number of daily contacts required to achieve an initial effective reproduction number of 1.2 inherently considers any non-pharmaceutical interventions in place at the beginning of the winter period in Europe prior to the emergence of Omicron. Seasonality is assumed to follow a cosine function, with a peak in seasonal infectivity occurring 6 weeks from model initialisation (representing mid-winter, Supplementary Figure 2).

2.2 Vaccine rollout

Four distinct risk groups are simulated for vaccination rollout; those 65 years of age and older or live with comorbidities (high-risk group), all other adults (18-64-year-olds), adolescents (12-17-year-olds), and children (5-11-years-olds). In our simulations, vaccinations start in the high-risk group in January 2021 reaching a 90% coverage rate on 1 December 2021. The 18-64-year-olds start vaccination on 1 May 2021, achieving 70% coverage on 1 December 2021. Adolescents 12-17 years of age started vaccinations on 1 October 2021 with a coverage of 50% achieved by 1 December 2021. Children 5-11 years of age start vaccinations of doses 1 and 2 on 1 December 2021 and are only included as part of the extended vaccination scenario. Vaccination groups, associated vaccination coverages as of 1 December 2021, and the simulated future scenarios are summarized in Supplementary Table 3.

2.3 Vaccine-induced immunity profile

We model an interval of 28 days between the first and second dose, and a maximum of 95% vaccine efficacy to be reached 14 days after the second dose (increasing with a sigmoidal curve). We assume that 90% of vaccination effect is transmission blocking (i.e. a 90% reduced probability to become infected after being exposed). We assume vaccinated individuals will always be administered two doses and assume that 95% of those vaccinated with doses one and two will accept a third-dose. Third-doses are administered 6 months after the second dose, after which the maximum vaccine efficacy of 95% is again reached 14 days after the last dose has been administered. One month after the last dose, immunity starts waning linearly to zero over

335 days[2,3]. The left panel of Supplementary Figure 1, titled 'immunity from vaccination', provides a schematic overview of the waning immunity pattern after vaccination.

2.4 Infection-induced immunity profile

After recovering from infection with SARS-CoV-2, individuals develop naturally acquired immunity with a maximum level of 90% reduced susceptibility which they maintain for a month, after which their immunity wanes linearly to zero over a period of 335 days[2,3]. The level of immunity is the reverse of the susceptibility of the individual, which thus increases over time. The Delta variant is assumed not to be immune evading, therefore immunity following infection from Delta and Omicron provides a similar risk of infection when exposed to Delta. The right panel of Supplementary Figure 1, titled 'immunity from natural infection', provides a schematic overview of the waning immunity pattern after natural infection.

2.5 Effect of variant properties and vaccination on prognosis

The individual's prognosis depends on multiple factors including age, vaccine status, co-morbidity, and variant severity. The probabilities of 1) a symptomatic case developing severe disease, 2) a severe case becoming critical, and 3) a critical case ultimately leading to death, are all defined as functions for the above-mentioned factors. For this study, we use probabilities reported in[4], updated to represent the additional risk of hospitalisation from infection with VOC Delta (B.1.617.2) [5–7]. In additional to age-related risk, the probability that an infected individual will develop severe symptoms is also scaled by the severity factor of the viral variant exposed to.

In this study we use a severity factor of 1 for VOC Delta (B.1.617.2), and consider a range of potential relative severity factors for VOC Omicron (B.1.1.529) between 0 and 2. That is, a variant that has 0%-200% severity of Delta. For vaccinated individuals that become infected (noting that the transmission-blocking action of the vaccine reduces the probability of infection), the probability of developing severe disease is reduced by the severity or disease-blocking property of the vaccine. The level to which the probability of severe disease is reduced is dependent upon the level of immunity at the time of infection.

Vaccine-induced immunity is assumed to wane over time and can be further decreased if

exposed to a variant with immune evading capacity. In this study, we consider the full range of potential immune evading properties of Omicron, from 0% to 100%. The probability that immunologically naïve (i.e., unvaccinated, and previously uninfected) individuals with no comorbidities infected with Delta (severity factor 1, 0% immune evading) develop severe disease are given in Supplementary Table 2.

3. Supplementary references

1. Le Rutte, E. A., Shattock, A. J., Chitnis, N., Kelly, S. L. & Penny, M. A. OpenCOVID plotting functions for 'Modelling the impact of Omicron and emerging variants on SARS-CoV-2 transmission and public health burden'. *Zenodo* (2022). doi:10.5281/zenodo.6532404

2. Wheatley, A. K. *et al.* Evolution of immune responses to SARS-CoV-2 in mild-moderate COVID-19. *Nat. Commun.* **12**, 1162 (2021).

3. Cohen, K. W. *et al.* Longitudinal analysis shows durable and broad immune memory after SARS-CoV-2 infection with persisting antibody responses and memory B and T cells. *Cell Reports Med.* **2**, 100354 (2021).

4. Shattock, A. J. *et al.* Impact of vaccination and non-pharmaceutical interventions on SARS-CoV-2 dynamics in Switzerland. *Epidemics* **38**, 100535 (2022).

5. Twohig, K. A. *et al.* Hospital admission and emergency care attendance risk for SARS-CoV-2 delta (B.1.617.2) compared with alpha (B.1.1.7) variants of concern: a cohort study. *Lancet Infect. Dis.* **22**, 35–42 (2022).

6. Sheikh, A., McMenamin, J., Taylor, B. & Robertson, C. SARS-CoV-2 Delta VOC in Scotland: demographics, risk of hospital admission, and vaccine effectiveness. *Lancet* **397**, 2461–2462 (2021).

7. (CDC), C. for D. C. and P. Risk for COVID-19 Infection, Hospitalization, and Death By Age Group. (2022). Available at: https://www.cdc.gov/coronavirus/2019-ncov/covid-data/investigations-discovery/hospitalization-death-by-age.html.
