# Supplementary material for: Modelling the impact of Omicron and emerging variants on SARS-CoV-2 transmission and public health burden
Source: Commun Med (Lond). 2022 Jul 25;2:93. doi: 10.1038/s43856-022-00154-z (PMC9311342; doi:10.1038/s43856-022-00154-z)
Supplement: Supplementary file 2 — Peer Review File [file 43856_2022_154_MOESM2_ESM.pdf]

nature portfolio

Peer Review File

Modelling the impact of Omicron and emerging variants on SARS-CoV-2 transmission and public health burden

Open Access This file is licensed under a Creative Commons Attribution 4.0 International License, which permits use, sharing, adaptation, distribution and reproduction in any medium or format, as long as you give appropriate credit to the original author(s) and the source, provide a link to the Creative Commons license, and indicate if changes were made. In the cases where the authors are anonymous, such as is the case for the reports of anonymous peer reviewers, author attribution should be to 'Anonymous Referee' followed by a clear attribution to the source work. The images or other third party material in this file are included in the article's Creative Commons license, unless indicated otherwise in a credit line to the material. If material is not included in the article's Creative Commons license and your intended use is not permitted by statutory regulation or exceeds the permitted use, you will need to obtain permission directly from the copyright holder. To view a copy of this license, visit http://creativecommons.org/licenses/by/4.0/.

This manuscript has been previously reviewed at another Nature Portfolio journal. This document only contains reviewer comments and rebuttal letters for versions considered at Communications Medicine

REVIEWERS' COMMENTS:

Reviewer #1 (Remarks to the Author):

The authors have addressed my prior concerns. I have no further comments.
